# *StreamSAXS*: a Python-based workflow platform for processing streaming SAXS/WAXS data

**DOI:** 10.1107/S1600577524005149

**Published:** 2024-07-15

**Authors:** Jiayi Wang, Zheng Dong, Yi Zhang, Wenqiang Hua, Zudeng Wang, Huilong Guo, Yiming Yang, Xiaoxue Bi

**Affiliations:** a Beijing Synchrotron Radiation Facility, Institute of High Energy Physics Chinese Academy of Sciences Beijing100049 People’s Republic of China; b Spallation Neutron Source Science Center, Dongguan523803, People’s Republic of China; c University of Chinese Academy of Sciences Beijing100049 People’s Republic of China; d Shanghai Synchrotron Radiation Facility, Shanghai Advanced Research Institute Chinese Academy of Sciences Shanghai201204 People’s Republic of China; e Global Energy Interconnection Group Co. Ltd, Beijing100031, People’s Republic of China; NSRRC, Taiwan

**Keywords:** X-ray scattering, data analysis, scientific workflow, plug-in framework

## Abstract

*StreamSAXS* is a Python-based small- and wide-angle X-ray scattering (SAXS/WAXS) data analysis workflow platform with graphical user interface (GUI). It aims to provide an interactive and user-friendly tool for analysis of both batch data files and real-time data streams. Users can easily create customizable workflows through the GUI to meet their specific needs. One characteristic of *StreamSAXS* is its plug-in framework, which enables developers to extend the built-in workflow tasks. Another feature is the support for both already acquired and real-time data sources, allowing *StreamSAXS* to function as an offline analysis platform or be integrated into large-scale acquisition systems for end-to-end data management. This paper presents the core design of *StreamSAXS* and provides user cases demonstrating its utilization for SAXS/WAXS data analysis in offline and online scenarios.

## Abstract 2

*StreamSAXS* has been developed as a Python-based desktop application within the SAXS/WAXS analysis system. It features a plug-in framework and seamless integration into large-scale acquisition systems for full-lifecycle data management.

## 1. Introduction

Recent advances in small- and wide-angle X-ray scattering (SAXS/WAXS) technologies, characterized by the emergence of highly brilliant synchrotron sources (Jiao *et al.*, 2018 ▸; Schroer *et al.*, 2018 ▸) and a diversity of experimental modes (Yaghmur & Hamad, 2022 ▸; Petersen & Weidenthaler, 2022 ▸; Hua *et al.*, 2024 ▸), have created the urgent requirement for data reduction and analysis software (Dong *et al.*, 2022 ▸). The main challenge is to facilitate automated data acquisition and customizable processing workflows, which can be lengthy and complex. The standalone program *Fit2D* (Hammersley *et al.*, 1996 ▸; Hammersley, 2016 ▸), developed by the European Synchrotron Radiation Facility (ESRF), was the pioneering data analysis software to attain worldwide recognition within the X-ray scattering and diffraction community. At certain synchrotron beamlines, the *Igor Pro*-based package *Nika* (Ilavsky, 2012 ▸) is also used for data reduction. In order to achieve further analysis like curve fitting or structural modeling, several well established programs such as *SASfit* (Breßler *et al.*, 2015 ▸), *GSAS-II* (Toby & Dreele, 2013 ▸), *SasView* (Doucet *et al.*, 2020 ▸), *ATSAS* (Petoukhov *et al.*, 2012 ▸; Manalastas-Cantos *et al.*, 2021 ▸), *GENFIT* (Spinozzi *et al.*, 2014 ▸) and *McSAS* (Bressler *et al.*, 2015 ▸) continue to be maintained. In response to the increased popularity of high-throughput experiments in recent years, ESRF has developed the *pyFAI* library (Ashiotis *et al.*, 2015 ▸; Kieffer *et al.*, 2020 ▸) in conjunction with Input/Output (I/O) library *FabIO* (Knudsen *et al.*, 2013 ▸) to surmount the speed constraints of *Fit2D*. Owing to the open-source nature and scientific community acceptance of Python, *pyFAI* can be readily incorporated into other software. Some programs with further analysis functions, tailored for SAXS/WAXS data, such as *BioXTAS RAW* (Hopkins *et al.*, 2017 ▸; Hopkins, 2024 ▸) and *DPDAK* (Benecke *et al.*, 2014 ▸), have been developed based on it.

To accommodate the long and diverse data processing pipelines anticipated in future SAXS/WAXS beamlines at the High Energy Photon Source (HEPS), China, a dynamically configurable and flexible workflow platform needs to be developed to orchestrate an automated and sequential data processing procedure. Workflow-driven systems complete operations in the form of tasks following user-defined processes, effectively enhancing flexibility and adaptability to changes in the processing flow. Due to the advantages in intelligent processing of large-scale data, workflow platforms can play a crucial role in scientific fields like medicine, materials and biotechnology (Kallio *et al.*, 2011 ▸; Yildiz *et al.*, 2019 ▸; Giardine *et al.*, 2005 ▸). There are also several examples of applying workflow platforms to synchrotron radiation facilities. The *DM* software (Veseli *et al.*, 2018 ▸), developed by the Advanced Photon Source, focuses on data movement, metadata cataloging, storage management and remote data access. The Java-based *DAWN* (Basham *et al.*, 2015 ▸; Filik *et al.*, 2017 ▸), created by Diamond Light Source, emphasizes providing a unified analysis platform for data from any synchrotron experiment. *DAWN* allows users to process experimental data at their home institution, but it currently does not support online processing. ESRF has designed a meta workflow system which provides both online and offline data access, *Ewoks* (Nolf *et al.*, 2022 ▸), to automate data processing and experiments. Among its ecosystem, *ewoksxrpd* (https://ewoksxrpd.readthedocs.io/) is a project specifically designed for SAXS/WAXS data processing through importing the *pyFAI* library, which utilizes the desktop graphical interface *Orange* (Demsar *et al.*, 2013 ▸) for visual programming. *EDNA* (Brennich *et al.*, 2016 ▸; Incardona *et al.*, 2009 ▸), which focuses on bioSAXS data, is another SAXS/WAXS data processing platform. It has a relatively fixed workflows and less human intervention, enabling easy assessment of experimental quality. Upon considering the easy integration into development ecosystem at HEPS such as deep binding with the Python-based data acquisition software framework *Mamba* (Liu *et al.*, 2022 ▸), using Python as the development language offers an advantage. Furthermore, many SAXS/WAXS data processing packages such as *pyFAI*, *LMfit* (Newville *et al.*, 2023 ▸) and *BioXTAS RAW* are developed in Python, which also offers platform independence. Therefore, using Python can simplify the process of encapsulation and integration of these packages within the system.

Looking ahead, large-scale SAXS/WAXS data necessitates greater reliance of scientific users on the software system provided by the beamline for the full-lifecycle management, encompassing data acquisition, analysis, visualization and storage. Therefore, there is an urgent need to develop online data processing software that seamlessly integrates with the data acquisition framework. With the data analysis component operating as a service within the acquisition framework, real-time data streams can be processed online for integrated results. Users could then evaluate data quality during acquisition to guide the subsequent scanning process. Moreover, advanced traditional algorithms or artificial intelligence algorithms can be further introduced to enhance scientific outcomes (Zhang, Dong*, et al.*, 2023 ▸; Zhang, Li*, et al.*, 2023 ▸; Zhao *et al.*, 2024 ▸).

In this work, a new workflow platform named *StreamSAXS* (see also Appendix *A*[App appa]) has been developed for SAXS/WAXS data processing using Python. Key features of *StreamSAXS* include: (i) customizable analysis tasks catering to user needs; (ii) visual definition of data flows between tasks via a graphical user interface for easy operation; (iii) open-source extensibility; (iv) integration with various existing libraries and algorithms; and (v) support for the analysis of real-time data streams. Consequently, *StreamSAXS* can serve as standalone software or be integrated into a large software framework to realize full-lifecycle management of data collection, processing and storage.

## 2. Platform design

*StreamSAXS* is a visual scientific workflow platform for SAXS/WAXS data reduction and analysis. Through the graphical user interface (GUI), scientists can create data analysis tasks and visually define the data flows between them, eliminating the need for programming. This alleviates users from the underlying execution and scheduling intricacies of the workflow, potentially reducing the barriers to data analysis and enhancing scientific productivity. In addition, the *StreamSAXS* platform provides task nodes supporting batch data from HDF5, TIF or TXT files, as well as real-time data streams from data acquisition systems. Developed in Python, the platform utilizes both the *PyQt* (https://pypi.org/project/PyQt5/) and *PyQtGraph* (https://www.pyqtgraph.org/) libraries. *PyQt*, a binding of the cross-platform GUI toolkit *Qt*, enables desktop application development using Python and is employed for creating the platform’s GUI. The pure-Python graphics library *PyQtGraph* is utilized for its high-speed performance, attributed to its heavy reliance on number crunching and *Qt*’s *GraphicsView* framework for rapid display. The following sections introduce the development of the *StreamSAXS* platform.

### 2.1. The GUI

The GUI of *StreamSAXS* includes a workflow widget for visually programming and constructing workflows, as well as plot widgets to display the processing results of task nodes within the workflow.

**Figure 1:**
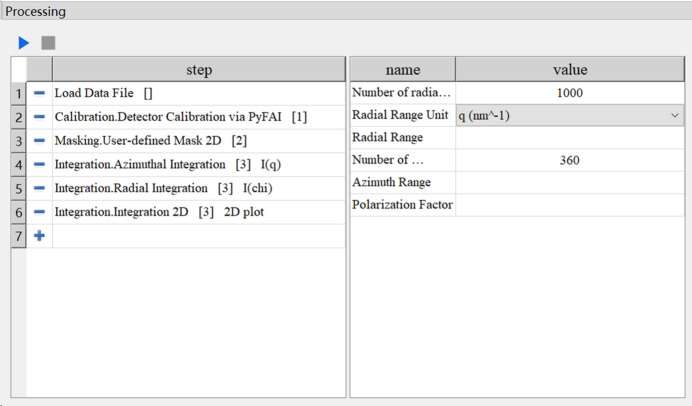
The workflow widget for *StreamSAXS*.

The *StreamSAXS* workflow widget employs a multilevel structure to establish a part-whole hierarchy among workflows, sub-workflows and individual tasks, thereby reducing the complexity of workflow construction. The workflow widget comprises two sections, as depicted in Fig. 1 ▸. The left section presents the names of each task node used in constructing each component of the workflow. Upon the addition of components to the widget, workflows are generated on-the-fly. By default, the platform automatically sets the input data flow of the new component from the output of the previous one. Hence, workflows are constructed with a list structure. Users can modify the input data flow of each component via point-and-click actions to alter structured workflows, simplifying the process to create on-the-fly workflows meeting analytical requirements. A component’s output can serve as input to multiple other components, while one component can also utilize multiple components as input. Therefore, the platform accommodates complex workflow structures like trees and graphs. Through the GUI, users can conveniently select SAXS/WAXS data processing algorithms and construct complex structures with features like parallel branching and directed acyclic diagrams. This enables simultaneous processing and displaying of results from multiple analyses. Besides processing results, the input, modification and presentation of parameters are also crucial in SAXS/WAXS data analysis. The right section of Fig. 1 ▸ displays parameters for the task node selected on the left. Prior to workflow execution, users populate analysis parameters. After execution, this widget displays the resulting parameter values from the currently selected component’s output in real time. All generated parameters can be stored along with analysis results according to the requirements. Additionally, *StreamSAXS* incorporates basic methods for saving and loading workflows, allowing for the storage of workflow structure and parameters for repeated usage. The saved workflows serve as configuration files, supporting text format browsing and editing.

For plot widgets, both the number and the layout can be customized by clicking and dragging. *StreamSAXS* provides real-time display widgets in the main window to plot the final results of a workflow. Meanwhile, various plot widgets can be linked to the task components, enabling real-time visualization of analyzed task node data. Five widget types were developed to visualize the data processing: (i) 1-D (one-dimensional) plot widget for displaying 1-D results like integral intensity profiles, fitting curves, Guinier plots and Porod curves; (ii) 2-D (two-dimensional) visualizer widget for displaying 2-D patterns such as raw scattering images, masked images and 2-D integral intensity maps; (iii) 2-D serial visualizer widget for displaying a series of 2-D patterns; (iv) 3-D (three-dimensional) slices visualizer widget for displaying arbitrary direction slices of 3-D data; (v) 2-D plot widget containing a series of final calculation values such as peak positions and gyration radii for user-defined workflows, shown in a 2-D mapping pattern using data coordinates. Users can opt to show specific task results based on needs, so the number of first four widgets is configurable. To facilitate mapping experiments, regions of interest (ROIs) can be extracted from the 2-D plot widget and the corresponding coordinate parameters directly feed into the scan sequence module of *Mamba* for further scanning procedures.

### 2.2. The plug-in framework

To accommodate flexible changes in SAXS/WAXS data processing and ensure software extensibility, *StreamSAXS* adopts a plugin framework to integrate diverse data processing methods on the platform. Plugins reside in a designated folder, with similar ones categorized together based on their processing methods. Each plugin is a class encompassing management options that instruct the core program on how to execute analysis, control functionality and handle plot visualization. Hence, developers can extend *StreamSAXS* by placing custom script files containing factory classes in the plugins directory. During *StreamSAXS* initialization, the platform searches the plugins folder within the application path for available plugins. Python’s reflection mechanism, MetaClass, automatically reads each discovered plugin class into the system. Every plugin is then automatically appended to the workflow widget, empowering users to effortlessly access corresponding task nodes in the GUI for constructing workflows.

**Figure 2:**
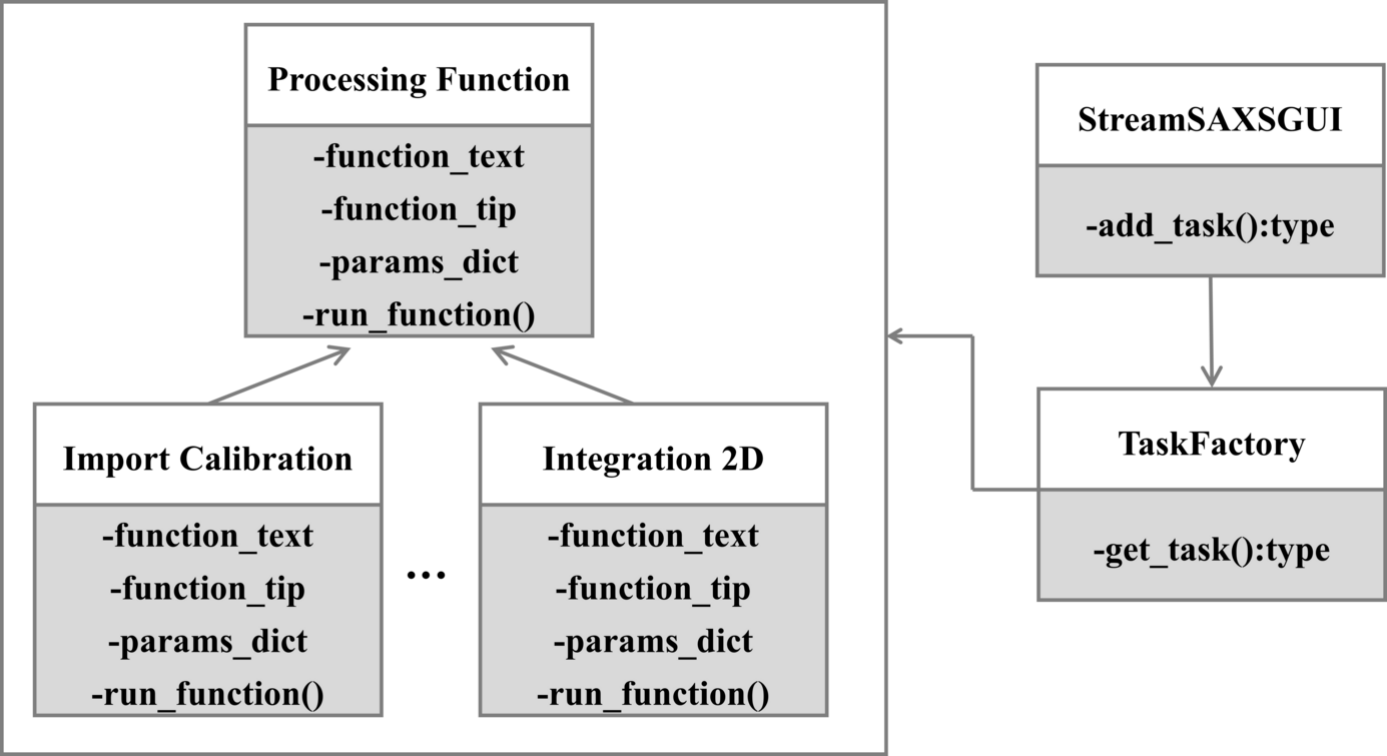
The Unified Modeling Language class diagram of the task node in *StreamSAXS*.

*StreamSAXS* implements plugins using the factory pattern (Cooper, 1998 ▸), a creational design pattern focused on object instantiation. The factory provides a unified interface to create objects without revealing the creation logic, offering flexibility in the development of data processing tasks. Since *StreamSAXS* is Python-based, abstract classes are utilized to define task object interfaces. Fig. 2 ▸ illustrates *StreamSAXS* constructing the abstract Processing Function class as the factory interface to meet the requirements for SAXS/WAXS data analysis tasks and visualization. The Processing Function class contains two static properties, function_text and function_tip, which respectively furnish the task node’s name and description for users. It also has a params_dict member to describe task parameters, and an abstract run_function method to execute the analysis process. Concrete data processing tasks are implemented by the overriding run_function. The ordered params_dict defines input parameter information like name, type, default value and tip. Supported data types include integer, float, string, tuple, Boolean and enumeration. The workflow widget renders parameter input and display based on the defined data types. The return value of run_function reflects the analysis result, containing data type, legend, axis details, title, line style and other plotting information. It also includes parameter data needed for workflow progression.

The framework assists developers in quickly encapsulating SAXS/WAXS analysis functions to construct workflow task nodes, and integrating them into the platform without concerning themselves with specific workflow execution details. As such, developers can concentrate exclusively on the analysis function itself, leaving the platform to handle workflow scheduling and task execution.

### 2.3. Data source of workflow

**Figure 3:**
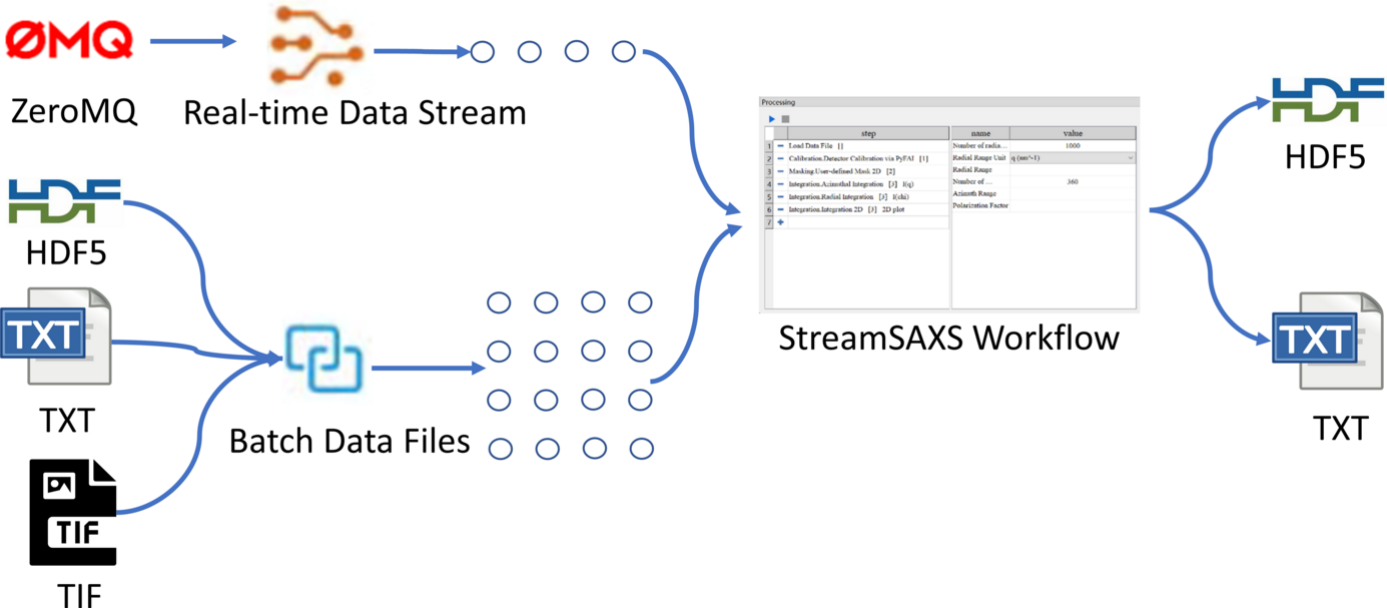
Two data source types for *StreamSAXS*.

Workflow construction in *StreamSAXS* begins with SAXS/WAXS data access. The platform supports two data source types as illustrated in Fig. 3 ▸: batch data files which are already acquired and real-time data streams from large-scale acquisition systems. HDF5, TIF and TXT formats are supported for file readings. Real-time data streams leverage the ZeroMQ (ZMQ) (https://zeromq.org/) network communication protocol. All data sources are mapped to the IO parameter type. The data source component can be built as needed and users access different sources by selecting appropriate parameters within the data source component. For instance, HDF5 sources require specifying the file location and dataset. Real-time ZMQ sources need the server’s IP address and port. Since ZMQ data are inherently represented as a dictionary, the key for the target data must also be provided. Therefore, beyond standalone usage for processing collected data files, *StreamSAXS* can also act as a client for any acquisition software with ZMQ transmission, facilitating online analysis and visualization.

## 3. User case

### 3.1. Long workflow

**Figure 4:**
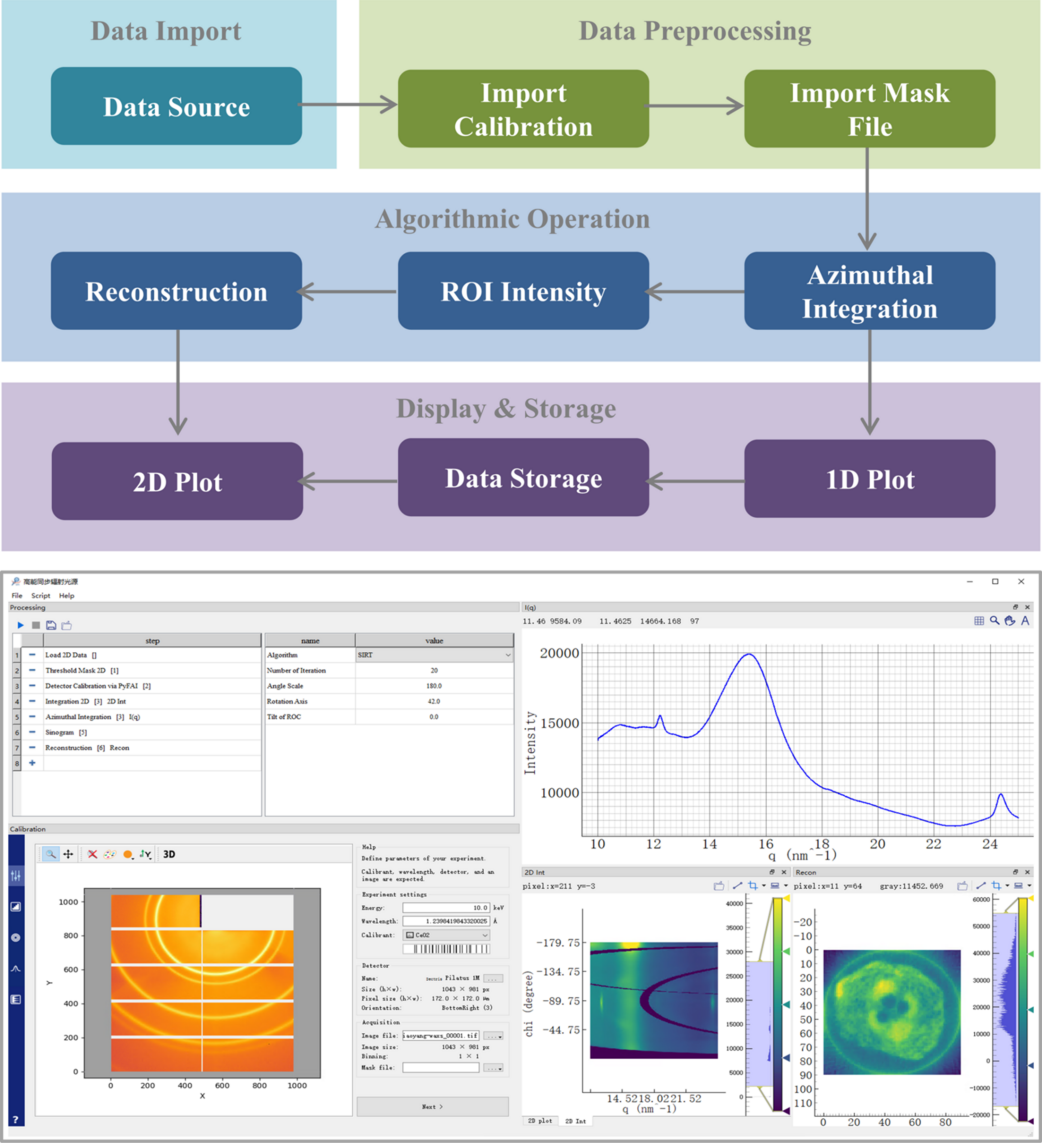
Workflow (top) and the GUI (bottom) for user case one. In the GUI, the upper left section is the workflow widget where a user-defined workflow for the WAXS tomography dataset of a bamboo sample has been configured. The lower left section is the graphical tool for geometry calibration inherited from *pyFAI*. The right half includes three types of plot widget: 1-D plot, 2-D visualizer and 2-D plot widgets.

In *StreamSAXS*, we have established a typical data analysis workflow (top of Fig. 4 ▸) to process the dataset obtained from a WAXS tomography experiment involving a bamboo sample, aiming to verify the platform’s capability (bottom of Fig. 4 ▸) in handling long processing pipelines. This customized workflow enables users to easily reconstruct the 3D hemicellulose content distribution within bamboo from the raw WAXS tomography dataset.

In detail, the whole processing steps are divided into four parts: (i) The first part includes the Data Source component. The data source for this case is a set of TIF files which are already acquired. (ii) The second part comprises the Import Calibration component and the Import Mask File component which are essentially standard for data preprocessing, and the detector geometric distortion correction is implemented during this preprocessing. We integrate the Calib2 calibration tools of *pyFAI* into *StreamSAXS* to complete the configuration process and then generate the configuration files including a calibration file and a mask file. Two configuration files are accessed through the file path in respective components. The configuration parameters are read into memory only once during workflow initialization and serve the entire data processing procedure, thus reducing file I/O operations and enhancing system performance. (iii) The third part involves the algorithmic operation crucial for the workflow. The Azimuthal Integration component converts 2-D masked images into 1-D azimuthal intensity profiles whose abscissa can be scattering vector, scattering angle or distance. The intensity uncertainty can also be calculated if needed by users. We integrate the Azimuthal Integrator module of *pyFAI* here to complete this regular integration process. This module can also be used to perform polarization correction. The ROI Intensity component extracts intensity summation within the selected region of a scattering curve. The following Reconstruction component achieves the 3-D reconstruction based on the input scalar. We provide back projection (BP), filtered back projection (FBP), iterative reconstruction technique (ART), simultaneous iterative reconstruction technique (SIRT), simultaneous algebraic reconstruction technique (SART) and conjugate gradient least squares (CGLS) algorithms to deal with the generated sinogram. In this workflow, the scattering peaks of hemicellulose were observed within the 1-D intensity profile and its peak intensity was further obtained by summation. The summed intensity here forms the sinogram for reconstruction, allowing for the successful reconstruction of the 3-D spatial distribution of hemicellulose based on a series of peak intensities in this tomographic experiment. (iv) The last part focuses on data display and storage. Any analysis results of the specified component can be displayed in real time as long as users establish the connection between component and display widget in the GUI, aiming to observe data trends and then assess data quality. Usually the final component of a workflow is Data Storage, which stores the processing results to a file. The platform supports both HDF5 and TXT file formats for data storage. Each data processing step is independent, allowing users to customize chunk storage based on their actual memory size. This means that processing results are periodically written from memory to the hard disk after each execution of a certain amount of data processing, thereby accommodating SAXS/WAXS data processing even on computers with limited performance. Additionally, during system execution, the task node generates essential processing parameters, and the system defaults to storing all initialization parameters and parameters generated during processing in a file.

### 3.2. Tree-structured workflow

**Figure 5:**
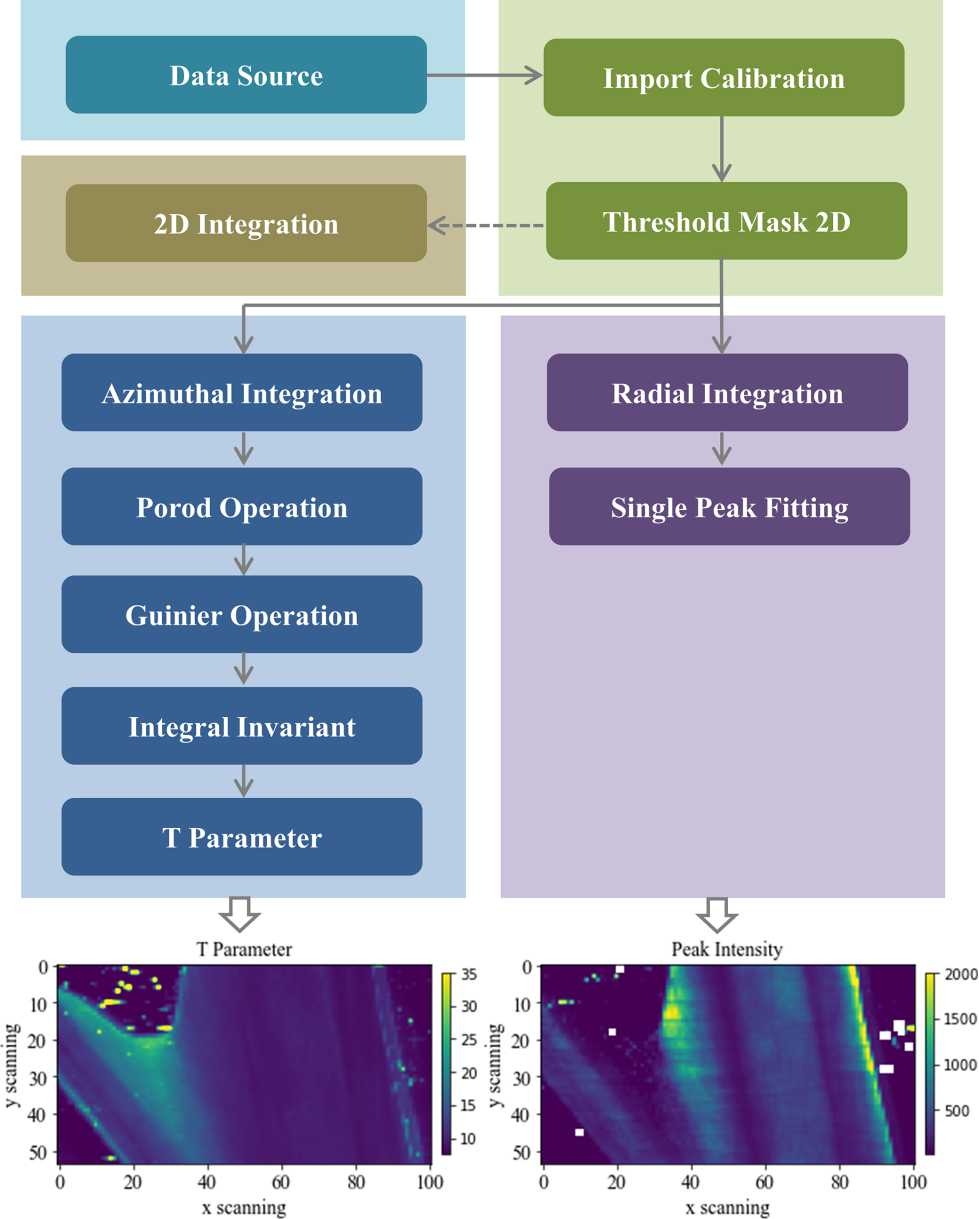
Tree-structured workflow for the scanning SAXS dataset of a mouse bone in user case two. Three parallel branches are included. One branch is 2-D integration which will be skipped during actual execution. Another branch utilizes azimuthal integration to obtain the T parameter. The third branch involves peak parameter extraction based on radial integration. The corresponding processing results are given at the bottom.

Fig. 5 ▸ shows a workflow applied to the scanning SAXS dataset for a mouse bone with a distinctive tree structure. The data source component is universal for all workflows. In the preprocessing part, the Background Subtraction component in which the transmission correction has been included is added before the Import Calibration component to exclude background scattering. And the mask is generated based on the threshold set within the Threshold Mask 2D component. Notably, the following flow expands into three parallel branches, allowing simultaneous analysis for different types of integration data. (i) One branch is the 2D Integration component calculating the azimuthal regrouped 2-D image. For some anisotropic samples, users may need to select an appropriate azimuth range for integration to analyze the strain and texture differences in different regions. Thus, 2-D integration can be operated to determine 1-D integration parameters. (ii) Another branch calculates the radial intensity profile (intensity–azimuth angle), and then leads to peak fitting, which reflects the distribution of collagen fibers through peak intensity. The Radial Integration component and the Single Peak Fitting component complete the above operations. (iii) The third branch focuses on analyzing the azimuthal intensity profile generated by the Azimuthal Integration component. The Porod Operation component and Guinier Operation component are used for Porod correction (Porod, 1952 ▸) and Guinier fitting (Guinier, 1939 ▸; Chen *et al.*, 2024 ▸), both essential for calculation of the integral invariant. Thus the Integral Invariant component will raise an error if either of the above two components is missing. Afterward, the T Parameter, an indicator of the volume-to-surface ratio, is determined. At present, the tasks within three parallel workflows are executed on a single core of the processor. The processing results given at the bottom of Fig. 5 ▸ are displayed in the GUI and stored by the Data Storage component which is omitted in the diagrammatic sketch. Benefiting from numerous Python libraries dedicated to SAXS/WAXS algorithms, several components, including Single Peak Fitting and Guinier Operation, are effortlessly executed using packages like *LMfit* and *BioXTAS RAW*. This implies that *StreamSAXS* can be extended with any existing libraries for further applications.

### 3.3. Integration with large-scale acquisition systems

A primary goal in developing *StreamSAXS* is enabling real-time data processing on streaming data directly ingested from acquisition systems at SAXS/WAXS beamlines. At HEPS, all beamlines will utilize the *Mamba* platform. Live streams from SAXS/WAXS detectors and metadata will be fed into *StreamSAXS* by the *Mamba* Data Worker (Li *et al.*, 2023 ▸) using the ZMQ message protocol. *StreamSAXS* processing results can then integrate with various control modules designed for *in situ* and scanning experiments to achieve feedback control. Presently, *StreamSAXS* is deployed at the 3W1 and 1W2A beamlines of the Beijing Synchrotron Radiation Facility (BSRF). Although handling *Mamba* streaming data is realized, currently *StreamSAXS* focuses on pure data processing as BSRF experiments are quite straightforward. Validating its role in fast online processing and feedback control still awaits commissioning of the new generation of SAXS/WAXS beamlines at HEPS.

## 4. Conclusion and future work

*StreamSAXS*, a desktop application within the SAXS/WAXS analysis system, allows scientists to seamlessly combine multiple signal processing tasks through an intuitive GUI. This user-friendly approach empowers scientists with limited programming skills to efficiently organize complex data processes. As a result, scientists can dedicate more of their attention to analyzing the processing results. Additionally, the platform features a plug-in framework that supports developers in secondary development efforts. This framework is both user friendly and easily extended, with plans for expansion to encompass diverse synchrotron radiation technologies, including imaging and spectroscopy. Furthermore, the system supports file-based data batch processing and real-time data analysis using data streams. As advanced light sources become more prevalent, scientific users increasingly rely on beamline-provided software systems throughout the entire data lifecycle, including acquisition, analysis, visualization and storage. This positions the platform for widespread adoption and continued development.

Looking ahead, our future development goals involve supplementing new data reduction tools (Shih *et al.*, 2022 ▸) and processing algorithms developed by beamline scientists or users, and integrating the workflow platform with distributed frameworks to enhance data analysis speed and reduce the run time of tasks with high time complexity. Moreover, we plan to expand the platform’s capabilities by incorporating various data transport protocols, such as *Kafka*, to further extend its versatility and functionality.

## Appendix A External libraries, language and availability

*StreamSAXS* is an open-source software distributed under the GPL v3 license. It can be download from the website https://github.com/dongzhengniall/streamSAXS, where documentation, mailing list, installation instruction and running examples are also given. *StreamSAXS* is currently compatible with Windows system and requires Python 3.7 to run. It also uses a number of third-party Python packages. These include *NumPy* (van der Walt *et al.*, 2011 ▸), *SciPy* (Virtanen *et al.*, 2020 ▸), *Matplotlib* (Hunter, 2007 ▸), *h5py* (https://www.h5py.org/), *PyQt5* (https://pypi.org/project/PyQt5/), *PyQtGraph* (https://www.pyqtgraph.org/), *pyFAI* (Ashiotis *et al.*, 2015 ▸; Kieffer *et al.*, 2020 ▸), *FabIO* (Knudsen *et al.*, 2013 ▸), *LMfit* (Newville *et al.*, 2023 ▸), *BioXTAS RAW* (Hopkins *et al.*, 2017 ▸), *OpenCV* (Bradski, 2000 ▸), *ASTRA* (van Aarle *et al.*, 2015 ▸), *filetype* (https://github.com/h2non/filetype), *Pillow* (https://pypi.org/project/pillow/), *tifffile* (Christoph, 2024 ▸), *PyYAML* (https://pypi.org/project/PyYAML/) and *skimage* (van der Walt *et al.*, 2014 ▸).
